# Postnatal development of the molecular complex underlying astrocyte polarization

**DOI:** 10.1007/s00429-014-0775-z

**Published:** 2014-04-29

**Authors:** Lisa K. Lunde, Laura M. A. Camassa, Eystein H. Hoddevik, Faraz H. Khan, Ole Petter Ottersen, Henning B. Boldt, Mahmood Amiry-Moghaddam

**Affiliations:** Laboratory of Molecular Neuroscience, Institute of Basic Medical Sciences, University of Oslo, Oslo, Norway

**Keywords:** Astrocyte polarization, Aquaporin-4, Extracellular matrix, Dystrophin associated protein complex, Brain development, Immunogold cytochemistry

## Abstract

Astrocytes are highly polarised cells with processes that ensheath microvessels, cover the brain surface, and abut synapses. The endfoot membrane domains facing microvessels and pia are enriched with aquaporin-4 water channels (AQP4) and other members of the dystrophin associated protein complex (DAPC). Several lines of evidence show that loss of astrocyte polarization, defined by the loss of proteins that are normally enriched in astrocyte endfeet, is a common denominator of several neurological diseases such as mesial temporal lobe epilepsy, Alzheimer’s disease, and stroke. Little is known about the mechanisms responsible for inducing astrocyte polarization in vivo. Here we introduce the term endfoot-basal lamina junctional complex (EBJC) to denote the proteins that consolidate and characterize the gliovascular interface. The present study was initiated in order to resolve the developmental profile of the EBJC in mouse brain. We show that the EBJC is established after the first week postnatally. Through a combination of methodological approaches, including light microscopic and high resolution immunogold cytochemistry, quantitative RT-PCR, and Western blotting, we demonstrate that the different members of this complex exhibit distinct ontogenic profiles––with the extracellular matrix (ECM) proteins laminin and agrin appearing earlier than the other members of the complex. Specifically, while laminin and agrin expression peak at P7, quantitative immunoblot analyses indicate that AQP4, α-syntrophin, and the inwardly rectifying K^+^ channel Kir4.1 expression increases towards adulthood. Our findings are consistent with ECM having an instructive role in establishing astrocyte polarization in postnatal development and emphasize the need to explore the involvement of ECM in neurological disease.

**Electronic supplementary material:**

The online version of this article (doi:10.1007/s00429-014-0775-z) contains supplementary material, which is available to authorized users.

## Introduction

A hallmark of astrocytes is their structural polarization. Most if not all astrocytes are equipped with endfeet that contact pia or brain microvessels, and with fine velate processes that abut on central synapses. These morphological characteristics were identified over 100 years ago, soon after astrocytes were first discovered (Somjen 1988). Only over the past few decades it was realized that the structural polarization of astrocytes reflects a distinct functional and biochemical compartmentalization. Notably, endfeet were found to exhibit much higher K^+^ conductance than other parts of the membrane, underpinning the concept of K^+^ siphoning (Newman et al. 1984). The fine perisynaptic astrocyte processes, on the other hand, were shown to harbor a distinct molecular profile mirroring their specific roles in transmitter uptake (Chaudhry et al. 1995).

The astroglial endfeet provide a near complete covering of cortical capillaries (Mathiisen et al. 2010) and contain a unique arrangement of proteins that sets them apart from other astroglial compartments. High resolution immunogold analyses (Amiry-Moghaddam and Ottersen 2013) indicate that endfeet membranes are enriched with a number of transporters and channels, including the inwardly rectifying K^+^ channel, Kir4.1 (Nagelhus et al. 1999); AQP4 (Nielsen et al. 1997), and the transient receptor potential vanilloid 4, TRPV4 (Benfenati et al. 2007). Kir4.1 is the main molecular correlate of the high K^+^ conductance in endfeet, while AQP4 confers a high water permeability on endfeet membranes. The endfoot pool of AQP4 represents an influx route of water in brain edema (Amiry-Moghaddam et al. 2003), but also has been implicated in physiological functions including water resorption postnatally (Haj-Yasein et al. 2011). In addition, recent data suggest that perivascular AQP4 might propel a paracapillary drainage route that helps clear the brain of β-amyloid and other molecules (Iliff et al. 2012). Two of the endfoot proteins (AQP4 and TRPV4) appear to form a molecular complex involved in osmosensing and regulatory volume control (Benfenati et al. 2011).

Loss of astrocyte polarization––defined as a loss of the proteins that normally are enriched in endfoot membranes––occurs in a number of pathophysiological and clinical conditions. Both AQP4 and Kir4.1 are reduced in CA1 endfoot membranes of patients with mesial temporal lobe epilepsy (MTLE) (Eid et al. 2005; Heuser et al. 2012). In an animal model of MTLE, loss of AQP4 was found to precede the development of chronic seizures (Alvestad et al. 2013). Further, the endfoot pool of AQP4 is strongly reduced after experimental stroke (Frydenlund et al. 2006) and in a mouse model of Alzheimer’s disease (Yang et al. 2011). Loss of astrocyte polarization will interfere with important homeostatic processes and clearance mechanisms and could thus contribute to the progress and symptomatology of diverse neurological conditions.

Given the importance of astroglial polarization in health and disease, a key question is how polarization is induced and maintained. Several lines of evidence indicate that dystrophin isoform DP71 and its associated proteins play a special role in this regard. The first data pointing to the involvement of a dystrophin associated protein complex (DAPC) came with studies of mice deficient in dystrophin (Frigeri et al. 2001; Vajda et al. 2002) or α-syntrophin (Neely et al. 2001).

α-Syntrophin is known to bind to DP71 as part of the DAPC. Mice deficient in dystrophin or α-syntrophin concurred in showing reduced expression of AQP4 in astrocytic endfeet (Frigeri et al. 2001; Neely et al. 2001; Amiry-Moghaddam et al. 2003). The dramatic loss of AQP4 observed after knockout of α-syntrophin suggested that the latter molecule could be the immediate anchor of AQP4. Biochemical analyses and co-immunoprecipitation experiments bolstered the idea that DAPC is expressed in endfoot membranes and essential for maintaining astrocyte polarization (Neely et al. 2001). It was proposed that the DAPC is kept in place primarily through its attachment to laminin and agrin, which are key constituents of the pericapillary basal lamina (Neely et al. 2001; Amiry-Moghaddam and Ottersen 2003; Guadagno and Moukhles 2004; Wolburg et al. 2011). This would explain why the level of AQP4 drops abruptly once the endfoot plasma membrane is reflected away from the basal lamina of brain microvessels (Nagelhus et al. 1999).

The present study was initiated in order to resolve the developmental profile of the DAPC and its binding partners––hereafter collectively termed the endfoot-basal lamina junctional complex (EBJC). Through a combination of methodological approaches, including high resolution immunogold cytochemistry (Amiry-Moghaddam and Ottersen 2013), we could show that the different members of this complex exhibit distinct ontogenetic profiles. The earliest proteins to appear were laminin and agrin, and these proteins reached peak levels as early as P7. In contrast, aquaporin-4 and the inwardly rectifying K^+^ channel Kir4.1 were weakly expressed at P7 and showed a sharp increase in expression towards adulthood. The present data provide new insight in the sequence of events that underlies the assembly of the gliovascular interface.

## Materials and methods

### Animals

Adult male and pregnant female C57BL/6 mice (Jackson Laboratories, Boulder, CO) were used in this study. The mice were allowed ad libitum access to food and drinking water. Animal experiments were performed according to the European Council law on protection of laboratory animals, with the approval of the University of Oslo’s Animal Care and Use Committee. Every effort was made to minimize the number of animals.

### Perfusion fixation

Animals from the postnatal groups (P0, P4, P7, P10, P13, P21) as well as adult (8 weeks) animals were anaesthetized with Equitisin (150 µl for adult mice) and transcardially perfused using 4 % formaldehyde (FA) and 0.1 % glutaraldehyde in 0.1 M phosphate buffer (PB) for at least 10 min, with an initial 20 s of ice cold 2 % dextran in 0.1 M PB. For immunofluorescence and peroxidase based immunocytochemistry, glutaraldehyde was omitted from the fixation solution. Brains were removed and postfixed in the fixation solution overnight and stored in a 1:10 dilution of the same solution in 0.1 M PB.

### Post-embedding immunogold electron microscopy

Brains were cut into 0.5–1.0 mm slices, different regions were dissected, cryoprotected, quick-frozen in liquid propane (−170 °C), and subjected to freeze substitution. Specimens were embedded in methacrylate resin (Lowicryl HM20) and polymerized by UV light below 0 °C. Ultrathin sections (70–100 nm) were cut using an ultratome (Reichert Ultracut S, Leica).

Immunogold labeling was carried as previously described (Yang et al. 2011; Promeneur et al. 2013). Briefly, The sections were rinsed in Tris-buffered saline with Triton X-100 (TBST, 5 mM Tris–HCl, 0.3 % NaCl, 0.1 % Triton X-100), incubated in 2 % human serum albumin, followed by primary antibody (AQP4) overnight, secondary antibody (15 nm gold) for 90 min, and contrasted with 2 % uranyl acetate for 90 s and 0.3 % lead citrate for 90 s. The sections were examined using a Tecnai 12 electron microscope at 60 kV.

### Immunogold quantitation

Quantitative analysis was performed as described elsewhere (Amiry-Moghaddam et al. 2004; Mathiisen et al. 2006). Briefly 19–20 digital images of capillaries or pial surface were acquired from each section. Linear densities of gold particles over astrocyte membranes were determined by an extension of analysis [Soft Imaging Systems (SIS), Münster, Germany]. Linear densities were determined semi-automatically and transferred to SPSS Version 16 (SPSS, Chicago, IL) for statistical analysis.

### Light microscopic immunocytochemistry

#### Immunofluorescence

The brains were cryoprotected using increasing levels of sucrose dissolved in 0.1 M PB (10–20–30 %), and snap frozen in optimal cutting temperature (OCT) compound on dry ice. Sagittal brain sections (14 µm) were collected on object glasses and stored at −80 °C.

For immunofluorescence the sections were rinsed with 0.01 M phosphate buffered saline (PBS) and permeabilized for 10 min with 0.1 % Triton X-100 in PBS. After blocking with 2 % bovine serum albumin (BSA) in PBS, the brain sections were incubated overnight with primary antibodies diluted in blocking solution. After washing in PBS, the brain sections were incubated with secondary antibodies diluted in blocking solution for 60 min. After a new wash in PBS, the brain sections were mounted using Prolong^®^ Gold antifade reagent with DAPI (Invitrogen, Molecular Probes). Confocal images were acquired using a Leica fluorescent microscope.

#### Immunoperoxidase

Cryoprotection was performed as described above. Cryoprotected brains were snap frozen in 30 % sucrose and free-floating sections (30 µm) were rinsed in PBS, exposed to 2 % H_2_O_2_ for 10 min to deplete endogenous peroxidase activity, followed by three rinses in PBS. All sections were left in double distilled H_2_O at 37 °C for 10 min prior to pepsin (Dako Ref S3002, 1 mg/mL 0.2 M HCl) exposure at 37 °C for 10 min, followed by three rinses in PBS, first of which for 15 min at 27 °C, thereafter at room temperature. Blocking was done using 2 % BSA in PBS-T (PBS, 0.1 % Triton X-100). The sections were then incubated in primary antibody diluted in blocking buffer overnight in cold room (4 °C), followed by three times rinse in PBS-T and 1 h incubation in secondary antibody in blocking buffer was followed by three times rinses in PBS-T. The sections were then incubated for 1 h with biotinylated-streptavidin horseradish peroxidase complex (GE healthcare UK limited, Rpn 1051 V) diluted 1:100 in PBS-T and rinsed in PBS-T × 3, followed by PBS × 3 and 0.5 mg/mL diaminobenzidine (3,3′-Diaminobenzidine Tetrahydrochloride 10 mg/tab, Sigma D5905, DAB) dissolved in 0.1 M PB for 5 min. Finally, sections were incubated for a titrated time to DAB solution with added 0.01 % H_2_O_2_ (1 min for Laminin and 1.5 min for agrin). The DAB/HRP reaction was stopped by 3 thorough rinses in 0.1 M PB. Thereafter, sections were mounted using glycerin gelatine and examined using a light microscope.

### SDS-PAGE and Western blotting

The mice (*n* = 3 for each age group) were anesthetized (Chloroform), decapitated and the brains were immediately frozen in liquid nitrogen and stored at −80 °C. Frozen mouse brains were homogenized in homogenization buffer (50 mM HEPES, 0.32 M Sucrose, 5 mM EDTA) using a mini-pestle (2 × 1 min). Immediately before homogenization, one tablet of [Protease Inhibitor Cocktail (PIC), Roche] was dissolved in 50 mL of the homogenization buffer. The supernatant of a 1,000 × g spin was used for immunoblotting. Protein concentrations were determined with a DC protein assay (Bio-Rad) according to the manufacturer’s instructions. SDS-PAGE and Western blots were carried out as described previously (Yang et al. 2011) with the following exceptions: 5–30 μg of protein (agrin: 2 μg, α-syntrophin: 15 μg, AQP4 (P0–P21): 15 μg, AQP4 (adult): 1 μg, β-dystroglycan: 5 μg, dystrophin: 10 μg, Kir4.1: 5 μg and laminin: 30 μg) was added per lane and separated by electrophoresis on 7.5, 10, 12.5 or 4–20 % polyacrylamide gels (Criterion Precast Gel, Bio-Rad) depending on the proteins examined.

For normalization, the membranes were washed several times in TBS-T [17 mM Trizma base (Sigma), 140 mM NaCl, 0.05 % Tween] following incubation with primary and secondary antibody as above.

Actin was used as a loading control. Membranes for which actin could not be used, Ponceau S staining was used instead (Aldridge et al. 2008; Romero-Calvo et al. 2010). This was the case for Kir4.1 and β-dystroglycan, both of which have MWs very close to that of actin, and in the case of laminin and agrin, which have MWs that are too high to be included in the same blots as actin (Aldridge et al. 2008; Romero-Calvo et al. 2010). Briefly, the membranes were incubated in Ponceau S stain for 3 min, washed until the colored protein bands could be visualized clearly and then scanned in a table top scanner at 600 dpi. The picture was analyzed in Photoshop using the Histogram function. The lane was marked with a thin stripe and the value was read off in the histogram. Linearity was obtained for Ponceau S staining as well as for actin controls.

### Quantitation

For the quantification of the bands in SDS-PAGE and Western blotting two methods were used:

1. Samples from each age were loaded and compared to a standard curve on the same membrane. The fluorescent signal was detected with a Typhoon 9410 Workstation (Typhoon 9410, Amersham Biosciences, Piscataway, NJ, USA) and quantified with a commercially available image analysis program (ImageQuantTM TL, Amersham Biosciences, Piscataway, NJ, USA).
2. All samples were loaded on the same membrane. The fluorescent signal was detected with a Kodak scanner (Kodak Image Station 4000 MM PRO) and quantified with a commercially available image analysis program (Kodak MI SE, Carestream Health, Inc., Rochester, NY, USA).

### Antibodies

For the complete list of primary and secondary antibodies used in this study see Table 1.

**Table 1:** Antibodies

Method	Primary antibody	Secondary antibody
Immunogold	AQP4, anti-rabbit, 1:500, Sigma-Aldrich	Goat anti rabbit 15 nm, 1:40, Amersham Bioscience
	Agrin, anti-rabbit, 1:500, Gift from Professor Markus A. Ruegg, University of Basel	Goat anti rabbit 15 nm, 1:40, Abcam
	Laminin (L9393), anti-rabbit, 1:100, Sigma-Aldrich	Goat anti rabbit 15 nm, 1:40, Abcam
Immunofluorescence	Agrin, anti-mouse, 1:100, Chemicon, Millipore, Med Probe	Alexa 488 donkey anti-mouse, 1:1,000, Molecular Probes
	α-Syntrophin (SYN259), anti-rabbit, 1 :200, Gift from Dr. Marvin E. Adams	Cy3 donkey-anti rabbit, 1:1,000, Jackson Immuno Research Laboratories, Inc.
	AQP4, anti-rabbit, 1:200, Chemicon, Millipore	Cy3 donkey-anti rabbit, 1:1,000, Jackson Immuno Research Laboratories, Inc.
	AQP4, anti-rabbit, 1:400, Sigma-Aldrich	Cy3 donkey-anti rabbit, 1:1,000, Jackson Immuno Research Laboratories, Inc.
	β-Dystroglycan (H-242), anti-rabbit, 1:200, Santa Cruz Biotech. Inc.	Cy3 donkey-anti rabbit, 1:1,000, Jackson Immuno Research Laboratories, Inc.
	CD 31 (PECAM-1), anti-rat, 1:800, BD Biosciences	Cy5 donkey-anti-rat, 1:1,000, Jackson Immuno Research Laboratories, Inc.
	Dystrophin (DP-71), anti-rabbit, 1:100, Abcam	Cy3 donkey-anti rabbit, 1:1,000, Jackson Immuno Research Laboratories, Inc.
Immunoperoxidase	Agrin, anti-rabbit, 1:1,000, Gift from Professor Markus A. Ruegg, University of Basel	Biotinylated donkey-anti-rabbit, 1:100, Pierce
	Laminin (L9393), anti-rabbit, 1:100, Sigma-Aldrich	Biotinylated donkey-anti-rabbit, 1:100, Pierce
Western blot	Agrin, anti-rabbit, 1:1,000, Gift from Professor P. Sonderegger	Anti-rabbit AP antibody, 1:10,000, Sigma-Aldrich
	α-Syntrophin, anti-rabbit, 1:1,000, Abcam	Anti-rabbit AP antibody, 1:10,000, Sigma-Aldrich
	AQP4, anti-rabbit, 1:1,500, Chemicon, Millipore	Anti-rabbit AP antibody, 1:10,000, Sigma-Aldrich
	β-Actin (A 2,066), anti-Rabbit, 1:500, Sigma-Aldrich	Anti-rabbit AP antibody, 1:10,000, Sigma-Aldrich
	β-Dystroglycan (H-242), anti-rabbit, 1:750, Santa Cruz Biotech. Inc.	Anti-rabbit AP antibody, 1:10,000, Sigma-Aldrich
	Dystrophin, anti-rabbit, 1:500, Abcam	Anti-rabbit AP antibody, 1:10,000, Sigma-Aldrich
	Kir4.1, anti-rabbit, 1:400, Alomone	Anti-rabbit AP antibody, 1:10,000, Sigma-Aldrich
	Laminin (L9393), anti-rabbit, 1:2,000, Sigma-Aldrich	Anti-rabbit AP antibody, 1:10,000, Sigma-Aldrich

### Quantitative real-time PCR

The mice (*n* = 4 for each age group) were anesthetized, decapitated and the brain was immediately frozen in liquid nitrogen and stored at −80 °C. Whole mouse brains were isolated, treated with RNA later (Ambion) and stored at −80 °C until further processing. Quantitative real-time PCR was carried out the same way as by (Boldt and Conover 2011) with the following exceptions. Total RNA was extracted using RNeasy Lipid Tissue kit (Qiagen). 5 mg of RNA was reverse-transcribed using oligo d(T)18 primers and RevertAid H Minus First Strand cDNA transcription reagents (Fermentas) in a reaction volume of 20 µl. The cDNA reaction mixture was diluted 100-fold in 10 mM Tris–HCl (pH 8.0), and real-time PCR was set up using 2 µl template in a reaction mixture of 20 µl containing AB Power SYBR Green PCR Master Mix (Applied Biosystems) and specific primers (200 nM) for amplification of target genes. Primers were designed using Primer3 software. Expression levels of target genes were assessed by absolute quantification. Real-time PCR was performed with plates using StepOnePlus real-time PCR system (Applied Biosystems). Amplicons (~200 bps) were generated in a two-step PCR (95 °C for 15 s, 60 °C for 60 s, 40 cycles). TATA box binding protein (TBP) was used as an endogenous control for normalization of gene expression. TBP was selected among several candidate housekeeping genes evaluated for this study because it was found to have the most constant level of expression across the developmental sample groups. The lower limit of detection was identified through amplification of targets known not to be expressed in mouse brain resulting in Ct values >32 corresponding to copy numbers <10 per ng total RNA.

For the complete list of primers see Table 2.

**Table 2:** Primer sequences for real time PCR

Gene	Forward	Reverse
*Agrn*	CAGTGGGGGACCTAGAAACA	ATGGCCAGAGCCATGTAGTC
*Aqp4*	TTTGGACCCGCAGTTATCAT	GTTGTCCTCCACCTCCATGT
*DagI*	CCGAGAAGAGCAGTGAGGAC	AGCTCATCCGCAAAGATGAT
*Dp71*	CAAGCTTACTCCTCCGCTCT	GAGCCTTCTGAGCTTCATGG
*Kcnj10*	TTATCAGAGCAGCCACTTCACC	TCTCTGTCTGAGTCGTCTGAC
*Lama1*	TGGATAAAGACAGGCCCTTG	ACTTTGGCACTGCTGATTCC
*Lama2*	ACCAGCCTACCTCCAGCTTT	CCCATTCCATCCATCTTCTG
*Snta1*	GCTGGCTGACAGAACAGTTG	TTCTGCATCATAGGGCACTG
*Tbp*	ACGGACAACTGCGTTGATTT	CAAGGCCTTCCAGCCTTATAG

### Statistical analysis

Obtained values were transferred to SPSS Version 16.0 (SPSS, Chicago, IL) and mean value of the groups were compared using ANOVA test. Data are presented as mean ± SE.

## Results

### Immunohistochemistry

**Fig. 1:**
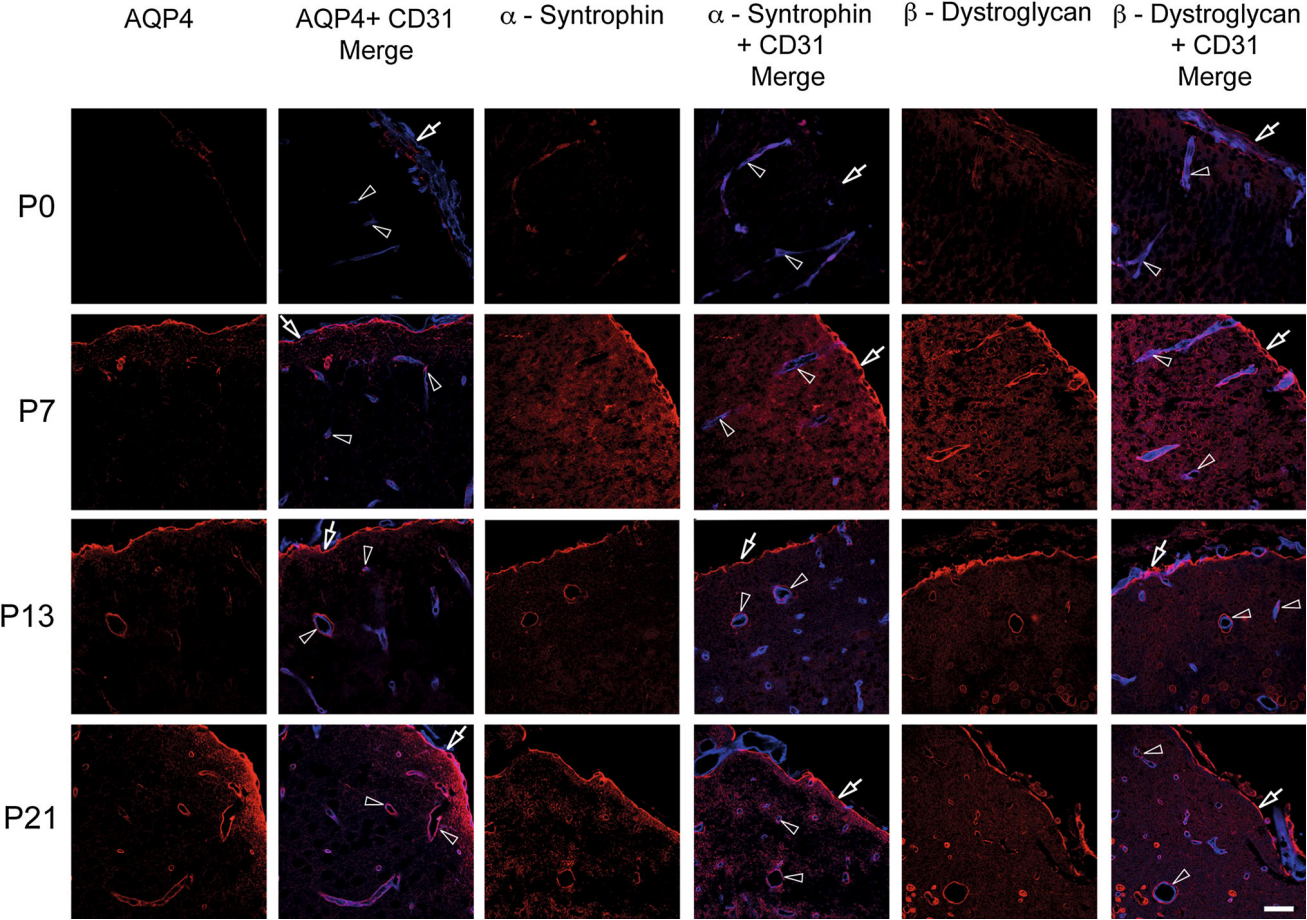
Members of the EBJC complex accumulate at brain surface and around vessels. Confocal images showing immunofluorescence labeling (*red*) of AQP4, α-syntrophin and β-dystroglycan in neocortex of postnatal mouse brain. These three members of the EBJC appear first at the brain surface and around penetrating vessels (lining the Virchow-Robins spaces), then around brain microvessels. Vessels (*arrowheads*) are identified by use of an endothelial marker (CD 31; *blue*). At P0, there was weak or no perivascular labeling for AQP4. At P7, AQP4 starts to appear around vessels (*arrowheads*), and at P13 and P21 there is AQP4 labeling around vessels of all calibers. Subpial AQP4 labeling (*arrows*) is present already at P0 and increases in strength towards P21. α-Syntrophin follows the same labeling pattern as AQP4. Weak labeling is observed for β-dystroglycan at P0. At P7, β-dystroglycan labeling is present at the brain surface (*arrows*) and around vessels of all calibers (*arrow heads*), thus being more extensive at this stage than the labeling for AQP4 and α-syntrophin. *Scale bar* 50 μm

The immunofluorescence analysis showed similar developmental profiles for AQP4, α-syntrophin, and β-dystroglycan (Fig. 1). These molecules are all members of the EBJC. None of these molecules were associated with the endothelial marker CD31 at P0. At P7, immunosignals for AQP4, α-syntrophin and β-dystroglycan were found subpially and around brain microvessels, with somewhat more prominent signals in the former site. β-Dystroglycan leads the other two molecules when it comes to accumulation around the endothelial lining (Fig. 1, second row, P7). At later developmental stages (P13 and P21) the diffuse neuropil labeling (pronounced for α-syntrophin and β-dystroglycan, less so for AQP4) subsides, leaving behind a rather distinct subpial and perivascular labeling. At P21, the subpial zone of immunofluorescence is broader for AQP4 than for the other members of the EBJC.

**Fig. 2:**
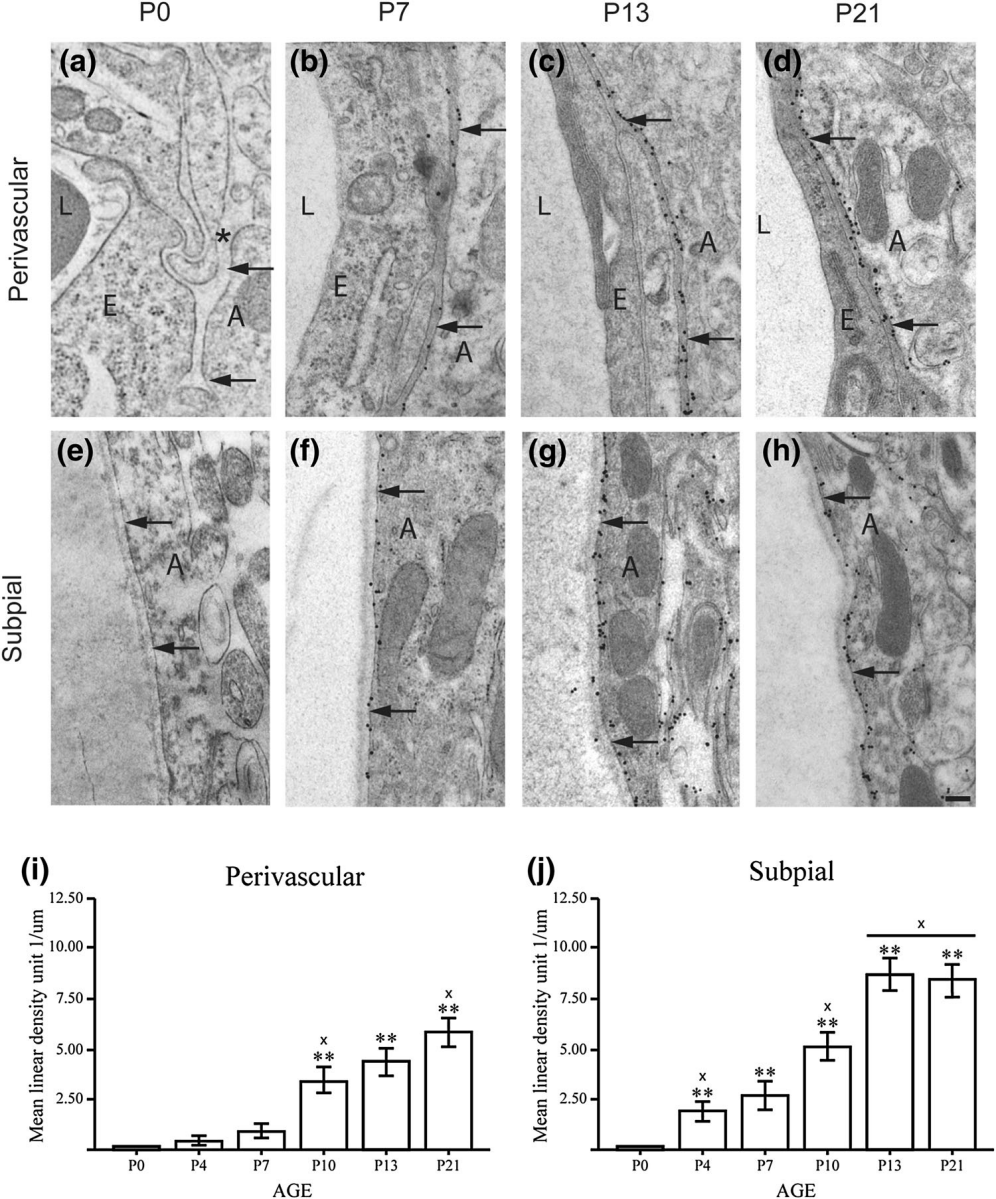
Immunogold analysis shows that subpial endfeet are the first to accumulate AQP4. **a**–**h** Postnatal immunogold labeling of AQP4 at the perivascular (**a**–**d**) and subpial (**e**–**h**) astrocyte membranes in mouse neocortex. Perivascular and subpial membrane domains are indicated by *arrows*. **a** At P0, the pericapillary basal lamina (*asterisk*) is immature, and there is no perivascular AQP4 immunogold labeling. **b** At P7, the basal lamina is distinct, and AQP4 appears in the perivascular membranes. **c**, **d** The AQP4 immunogold density increases further at P13 and P21. **e**–**h** The subpial basal lamina is well developed already at P0, and is associated with distinct AQP4 immunogold labeling in subpial astrocyte membranes. At P13 and P21, the AQP4 labeling extends into the glial lamellae beneath the pial surface. *E* endothelial cells, *L* vessel lumen, *A* astrocyte (*Scale bar* 200 nm). **i**, **j** Quantitative analysis of AQP4 immunogold labeling in perivascular (**i**) and subpial (**j**) membranes. At P4 and P7, the linear density of gold particles (no. of particles per µm membrane) is higher in subpial membranes than in perivascular ones. **Significantly different from P0; ‘x’ significantly different from previous value. *Error bars* indicate ±2 SE, *p* = 0.05

Immunogold cytochemistry confirmed and extended the immunofluorescence analysis. AQP4 could not be detected at P0, when the basal lamina was indistinct, but appeared at P4 with stronger signals subpially than perivascularly (Fig. 2). The immunogold signal for AQP4 increased from P4 to P21 (Fig. 2). AQP4 labeling of non-endfoot membranes occurred primarily in the subpial zone (particularly pronounced in Fig. 2g, h; Supplementary Fig. S1), as predicted from the immunofluorescence data (above).

**Fig. 3:**
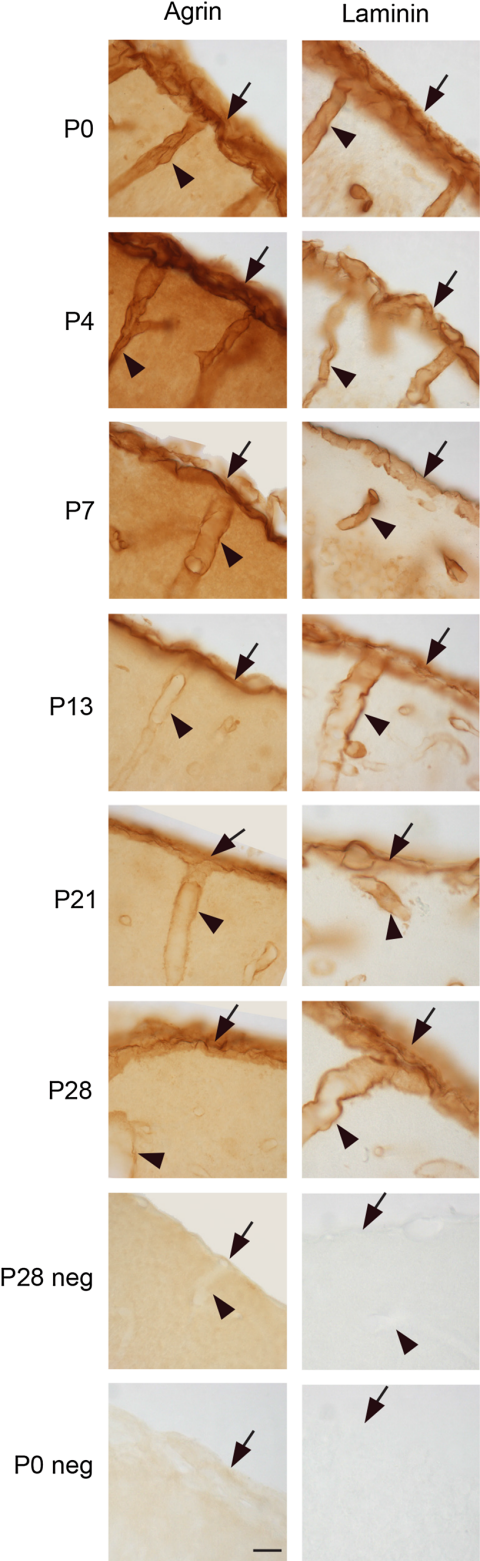
Agrin and laminin are present already at P0. Light microscopic pictures showing immunoperoxidase labeling of agrin (*left column*) and laminin (*right column*) in postnatal mouse neocortex. Agrin and laminin are strongly expressed at the pial surface (*arrows*) and around vessels (*arrow heads*) at P0. The labeling for both molecules becomes weaker towards P28. Labeling is removed by omission of primary antibodies (lower 2 rows; sections from P28 and P0, respectively). *Scale bar* 100 μm

In contrast to AQP4, α-syntrophin, and β-dystroglycan, the two basal lamina molecules agrin and laminin were strongly expressed already at P0 (Fig. 3). Both molecules occurred at the pial surface as well as perivascularly, and showed stronger DAB immunosignals at P7 than at later postnatal stages. The perivascular labeling, in particular, appeared to abate with age.

**Fig. 4:**
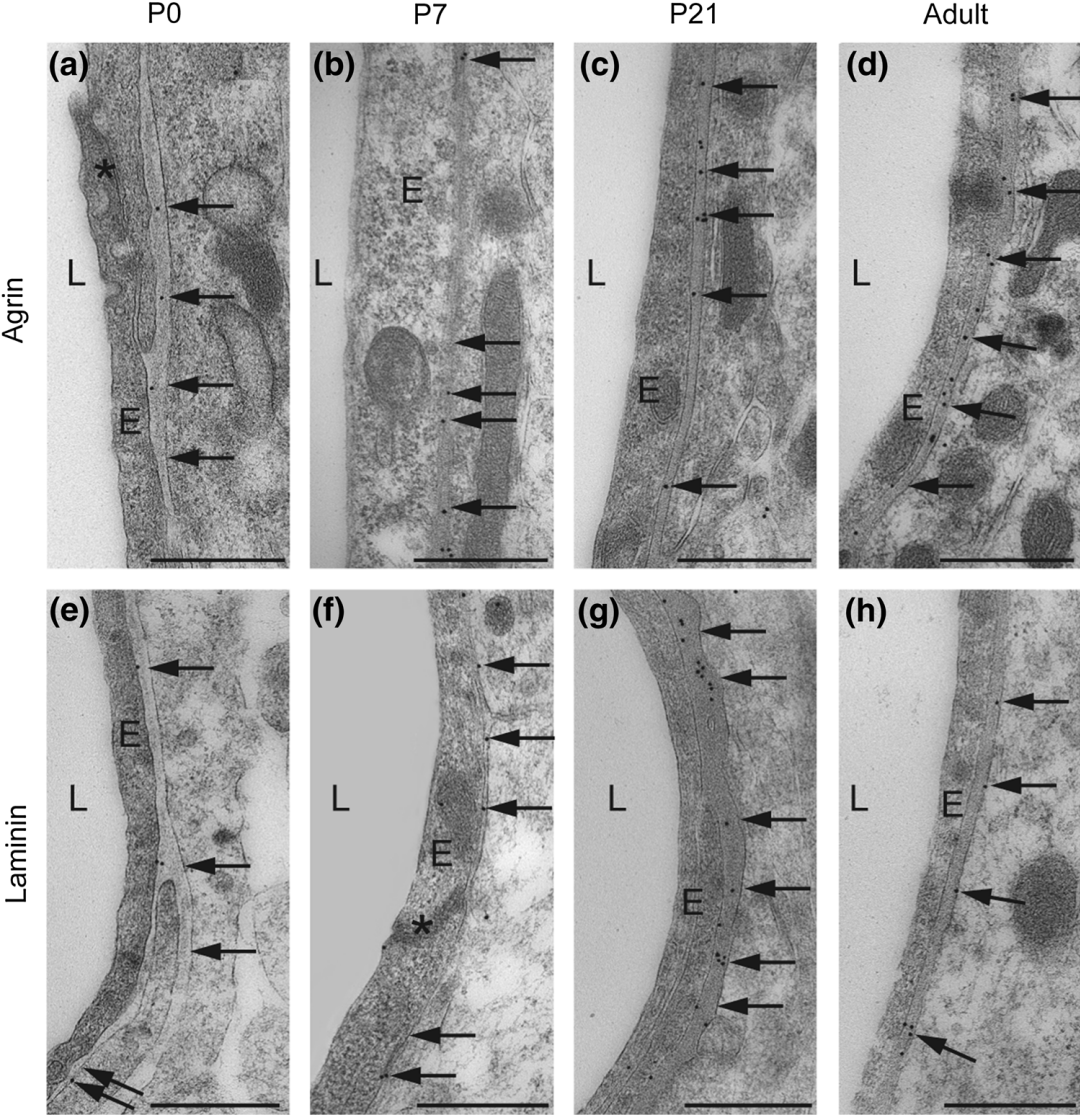
Agrin and laminin are confined to the perivascular basal lamina. Immunogold labeling confirms localization of agrin (**a**–**d**) and laminin (**e**–**h**) to the perivascular basal lamina (*arrows*). Both proteins are present throughout the postnatal period. Electron micrographs of postnatal mouse neocortex. *E* endothelial cells, *L* vessel lumen, *asterisk* tight junction. *Scale bar* 0.5 μm

**Fig. 5:**
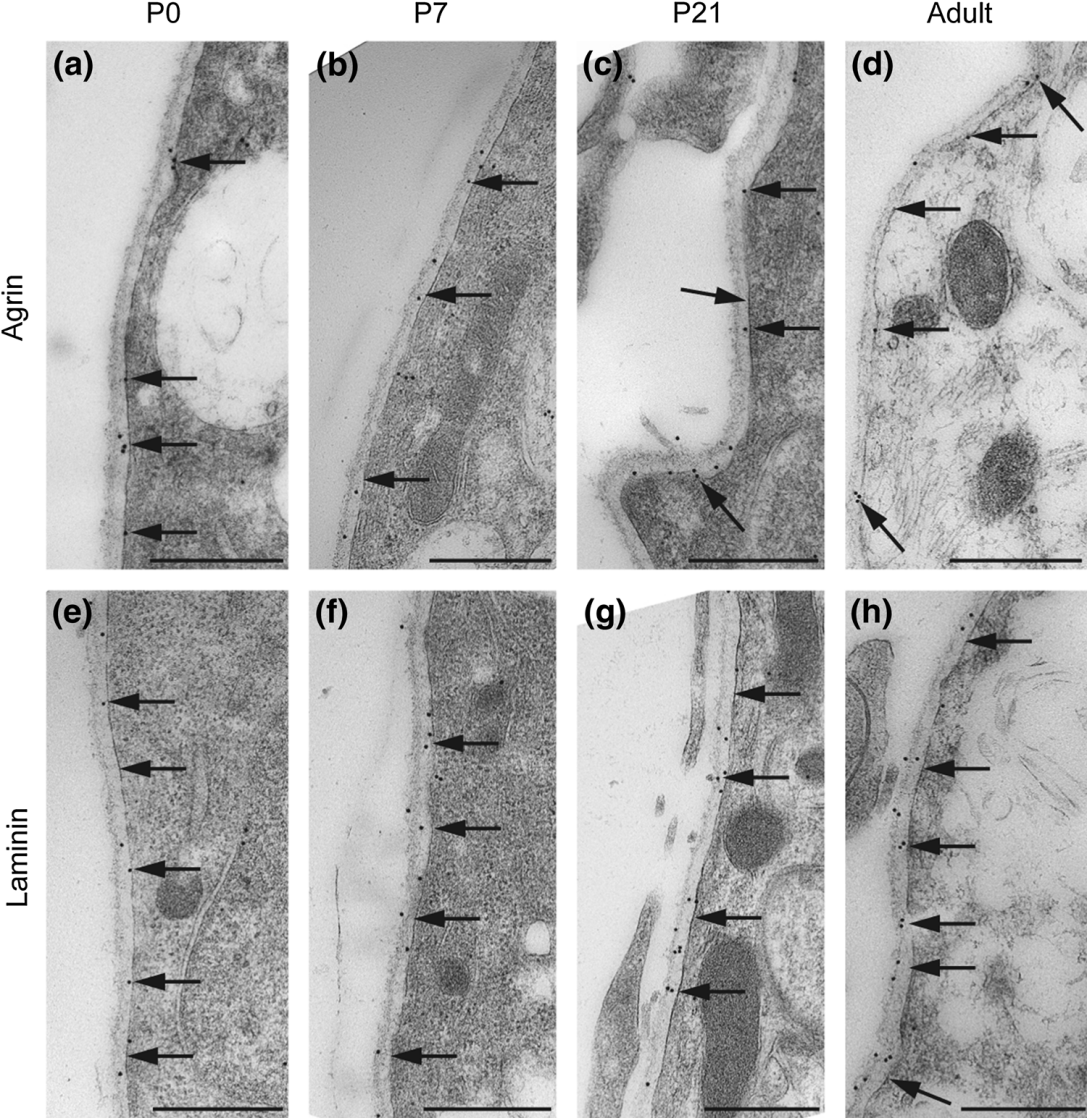
Agrin and laminin also occur in subpial basal lamina. Electron micrographs of immunogold labeling of agrin (**a**–**d**) and laminin (**e**–**h**). Both proteins are present in the basal lamina (*arrows*) opposed to subpial astrocyte endfeet. Labeling is distinct already at P0 and persists throughout the postnatal period. *Scale bar* 0.5 μm

To verify that the DAB signal represented laminin and agrin in the appropriate location (i.e., in the basal lamina, consistent with their being members of the EBJC), an immunogold analysis was performed. Immunogold particles for agrin and laminin were superimposed on the perivascular and subpial basal laminae as early as P0 (Figs. 4 and 5, respectively).

The specificity of the antisera was confirmed by using knockout animals in case of AQP4, α-syntrophin and β-dystroglycan (not shown). The antibody to agrin has been tested previously on agrin knockout mice (Stephan et al. 2008). Knockout lines are not available for laminin. The precise localization of lamin and agrin immunosignals to the basal lamina (Figs. 4 and 5) indicates absence of unspecific labeling. Labeling was abolished after omission of primary antibodies, ruling out nonselective binding of the secondary antibody.

### Quantitative Real-Time PCR

**Fig. 6:**
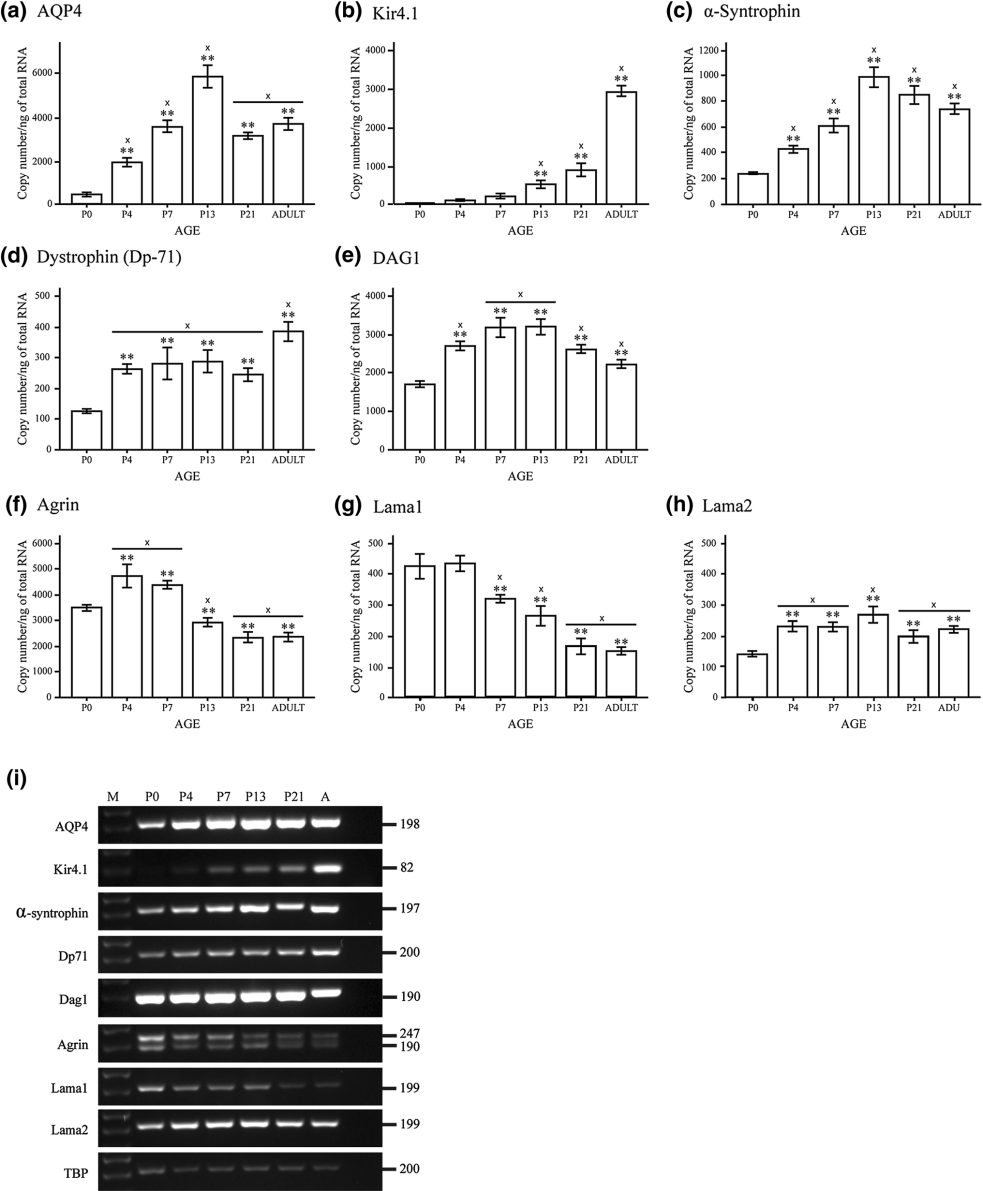
Different members of the EBJC complex have different mRNA signatures during development. **a**–**h** Quantitative real time PCR analysis of mouse brains at different stages of development. *Graphs* illustrate the copy number of different mRNA species, compared to the total amount of RNA (ng). The different EBJC members segregate in several groups, in regard to the developmental profile of their respective mRNAs. mRNAs encoding AQP4 and α-syntrophin are weakly expressed at birth and peak before adulthood, while mRNAs encoding agrin and Lama1 are abundant at birth with decreasing levels towards the adult stage. The remaining mRNA species show a rather stable expression throughout postnatal development (dystrophin, Dag1, Lama2) or a sharp increase in expression towards adulthood (Kir4.1). Dag1 encodes both α- and β-dystroglycan. **Significantly different from P0 and ‘x’ significantly different from previous value. *Error bars* indicate ±2 SE, *p* = 0.05. **i** Representative DNA agarose gel electrophoresis showing the EBJC expression profile during postnatal development of mouse brain. PCR products were generated using representative cDNA samples from developmental stage P0, P4, P7, P13, P21 and adult (*A*) as indicated above each lane on the gel. A DNA marker (*M*) was included in the first lane. The EBJC sample set includes from top to bottom: AQP4, Kir4.1, α-syntrophin, Dp71, Dag1, agrin, Lama1, Lama2 and TBP. The latter was included to verify equivalent amounts of cDNA template across samples in the 30-cycle endpoint PCRs using GoTaq Green polymerase (Promega) and specific primers (Table 2). PCR product sizes in base pairs are shown to the *right* of each gel insert. Two bands are visible in the gel insert for agrin, which is explained by co-expression of two mRNA species resulting from alternative splicing

The molecules under investigation formed two distinct groups in regard to the developmental profile of their respective messengers (Fig. 6). mRNAs encoding AQP4 and other members of the dystrophin complex (α-syntrophin, β-dystroglycan, and the dystrophin isoform DP71) were scarce at P0 and increased in abundance towards a distinct peak at P13 (AQP4 and α-syntrophin) or a broader peak at P7–P13/21 (β-dystroglycan and DP71). DP71 and Kir4.1 (both being members of the DAPC) stood out as the only molecules whose messengers continue to increase until adulthood.

The second group of mRNAs (i.e., those encoding laminin and agrin) showed entirely different developmental profiles. Agrin-mRNA and Lama1 (encoding the α1 isoform of laminin) were more abundant at early postnatal stages than at later stages in development (Fig. 6f, g). Both mRNAs peaked near P4. Lama2 (encoding the α2 isoform of laminin) was relatively weakly expressed at P0 and peaked at P13. Lama2 differed from Lama1 by showing a rather stable expression level towards adulthood (Fig. 6g, h).

### Immunoblots

**Fig. 7:**
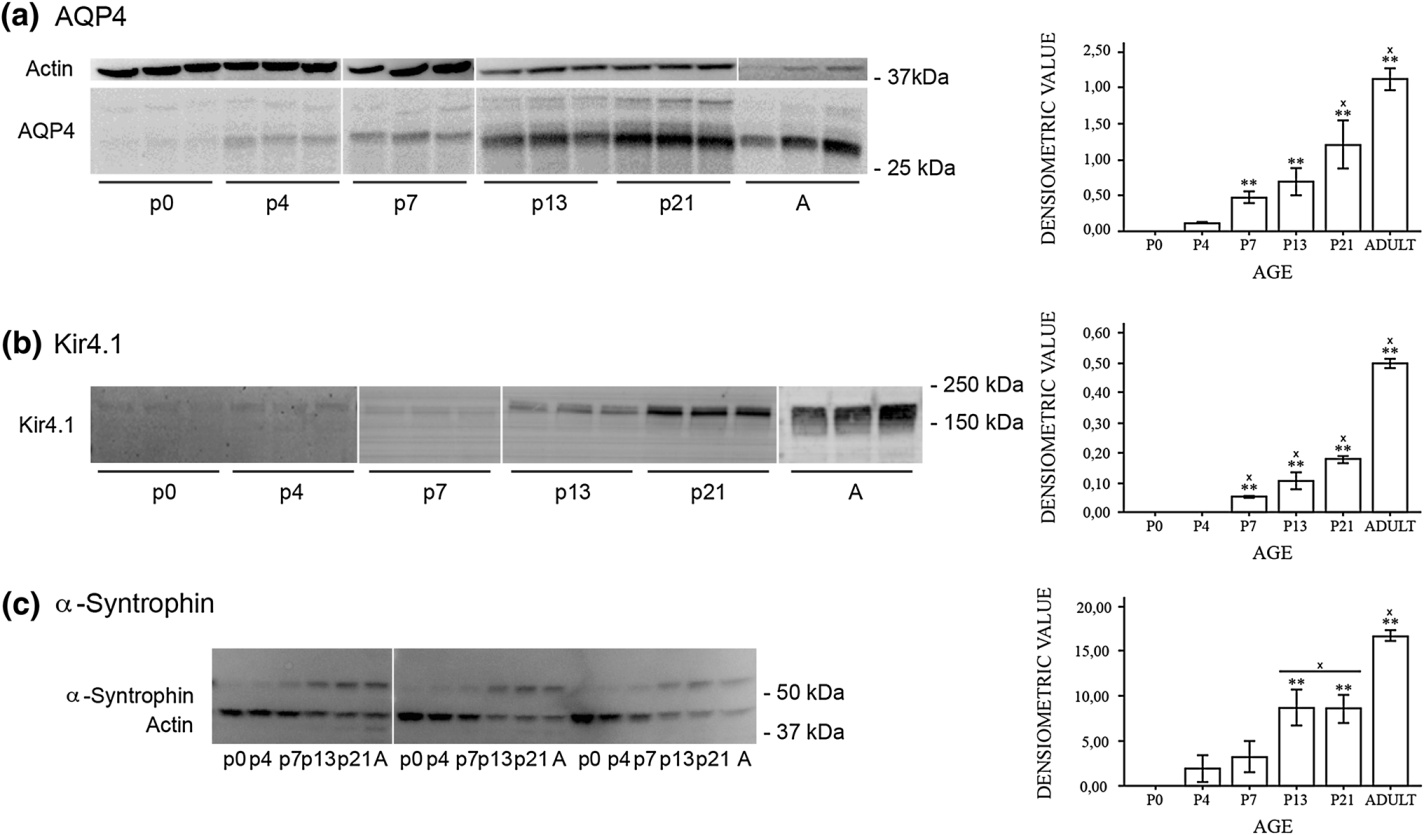
AQP4, Kir4.1, and α-syntrophin increase towards adulthood. Western blots of whole brain homogenates from the postnatal day 0 to 21 (P0–P21) and adult (*A*) mice. Representative immunoblots for AQP4, Kir4.1, and α-syntrophin (*left panels*) and corresponding quantitation (densitometric values; right panels). **a** The AQP4 antibody labelled two bands at about 30 kDa corresponding to the M1 and M23 isoforms of AQP4. A third band around 35 kDa was not included in the quantitative analysis. β-Actin was used as loading control. The densitometric analysis revealed an increasing immunosignal for AQP4 protein in the postnatal period. **b** Immunoblot of Kir4.1 revealed a major band at ≈200 kDa which corresponds to the tetrameric form of Kir4.1 (Connors and Kofuji 2006; Olsen et al. 2006). Ponceau red staining was used as loading control (not shown). The developmental pattern mimics that of AQP4 (**a**). **c** Immunoblot of α-syntrophin revealed a major band at 59 kDa and a weaker band at slightly higher molecular weight. The major band––absent from α-syntrophin knockout brains––was used for quantitative analysis. β-Actin was used as loading control. The expression pattern for α-syntrophin was similar to those of AQP4 and Kir4.1. **Significantly different from P0 and ‘x’ significantly different from previous value. *Error bars* indicate ±2 SE, *p* = 0.05

**Fig. 8:**
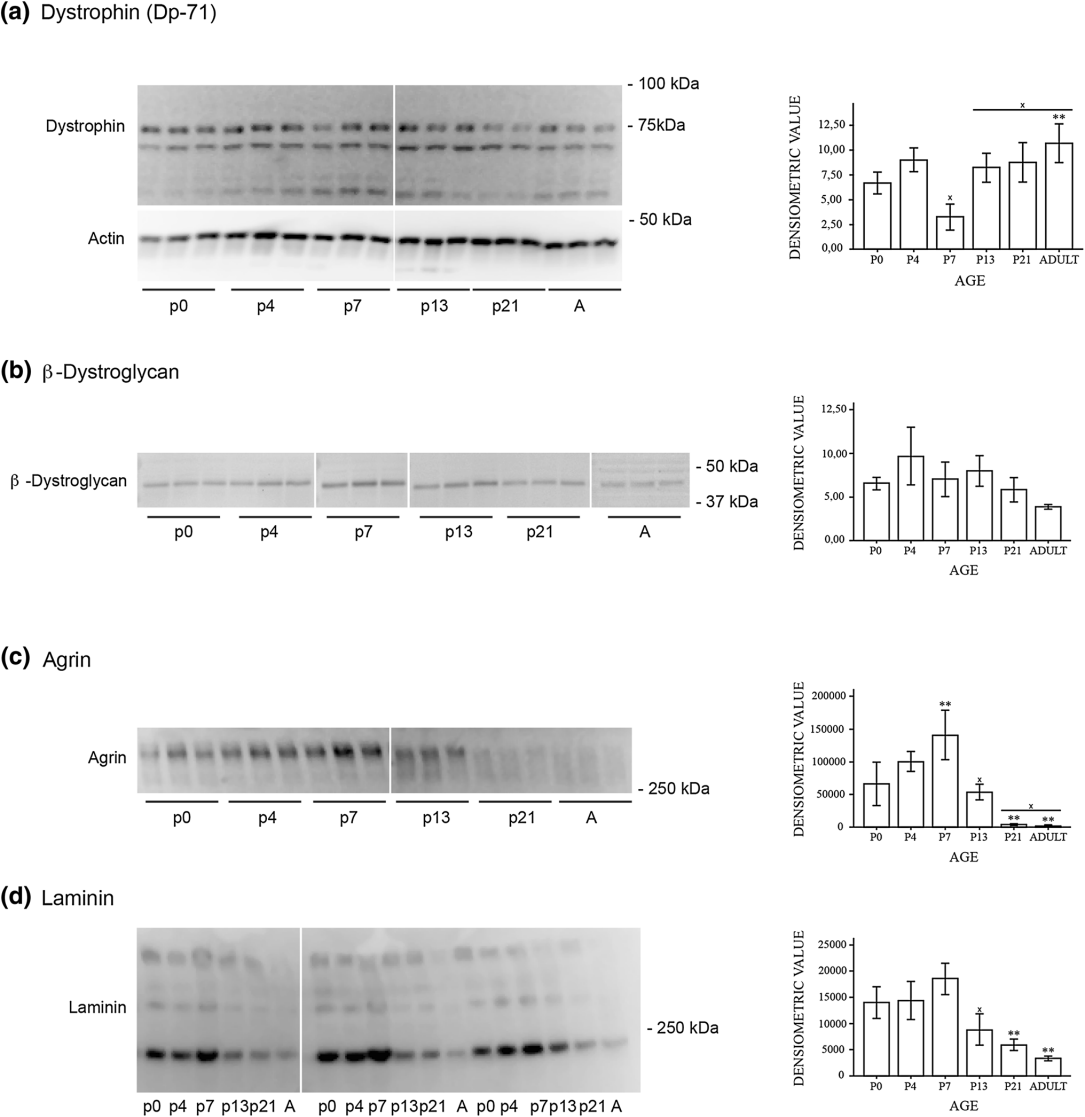
Agrin and laminin decrease with postnatal age. Western blots of whole mouse brain homogenate. Representative immunoblots of DP71, β-dystroglycan, agrin and laminin at different postnatal ages and adult (*A*) mice (*left panels*). The immunoblots were subjected to densitometric analysis (*right panels*). **a** Immunoblot of DP71 revealed a band at ≈71 kDa. There were also bands at ≈65 kDa and ≈55 kDa which could represent other dystrophin isoforms or degradation products. β-Actin was used as loading control. The immunosignal for DP71 was rather stable in the postnatal period. **b** Immunoblot of β-dystroglycan revealed a major band at ≈42 kDa. Ponceau red staining was used as loading control (not shown). The protein level is stable in the postnatal period. **c** Immunoblot of agrin revealed a major band at ≈300 kDa as shown previously (Stephan et al. 2008). Ponceau red staining was used as loading control (not shown). The immunosignal for agrin reached a peak at P7 (significantly higher than P0) with a sharp decline towards the adult level. **d** Immunoblot of laminin with whole brain homogenate revealed a major band at ≈200 kDa and two weaker bands at ≈400 kDa and ≈600–700 kDa. The 200 kDa band corresponds to the β- and γ-chain of laminin while the 400 kDa band corresponds to the α1-chain (Zhang et al. 2007). Ponceau red staining was used as loading control (not shown). Quantitative analysis of the 200 kDa band shows a pattern similar to that of agrin. Quantitation of the other two bands revealed no significant differences between the different postnatal stages. **Significantly different from P0 and ‘x’ significantly different from previous value. *Error bars* indicate ±2 SE, *p* = 0.05

The molecules under study segregate in three groups in regard to their expression at the protein level (Figs. 7 and 8). AQP4 mirrored α-syntrophin and Kir4.1 in showing a continuous increase from being close to undetectable at P0 to being strongly expressed at adult stages. DP71 and β-dystroglycan, on the other hand, are rather stable throughout postnatal development. Laminin and agrin formed a third group that peaked at P7 and thereafter displayed a sharp decline towards adulthood.

## Discussion

Evidence is accruing that the EBJC plays a central role in brain function and neurological disease. Hence, the integrity of the EBJC is a *conditio sine qua non* for maintaining the functional and biochemical polarization of astroglial cells. By orchestrating the molecular organization of the end-foot membranes of astrocytes, the EBJC serves a role akin to that of the protein complex that dictates the organization of pre- and postsynaptic membranes of neurons.

While the properties and developmental profiles of proteins responsible for neuronal polarization have been subjected to intensive studies (Arimura and Kaibuchi 2007) much less is known about the proteins of the EBJC. Notably, the sequence by which the different EBJC proteins appear and reach peak expression has not been investigated in detail. Insight in the developmental profile of the EBJC is likely to inform our understanding of the mechanisms that underpin the organization of the gliovascular interface and of the glia limitans at the brain surface. The gliovascular interface attracts particular interest in the context of cerebral blood flow regulation (Iadecola and Nedergaard 2007) while new data indicate that glia limitans is specifically implicated in volume control (Papadopoulos et al. 2004; Benfenati et al. 2011).

Unravelling the developmental profile of the EBJC requires a high anatomical resolution that matches the spatially confined expression of this junctional complex. At the same time, there must be possibilities for quantitation in order to accurately determine the peak of expression. Post-embedding immunogold procedures are uniquely suited to the task at hand. The lateral resolution of this procedure is in the range of ~30 nm, which compares favorably with the lateral dimension of the endfeet (in the order of 1000 nm) and the thickness of the basal lamina (in the order of 100 nm). Gold particles are easily identified and their number bears a well-defined relation––often close to linear (Mathiisen et al. 2006; Amiry-Moghaddam and Ottersen 2013)––to the number of target proteins. To the best of our knowledge this study represents the first successful immunogold application in brain of antibodies to pan-laminin and agrin. The basal lamina proteins laminin and agrin were the first proteins to appear at the gliovascular interface, according to the present analysis. Both proteins were detected at P0, predating the appearance of AQP4. However, a possible caveat needs to be considered: the threshold for detection might differ between different proteins. Thus, proteins may be differentially affected by steric hindrance or by the relatively harsh conditions that prevail during tissue preparation for immunogold cytochemistry. It is of note that the freeze substitution approach currently applied is more “antigen friendly” than traditional procedures for tissue preparation (Amiry-Moghaddam and Ottersen 2013). Also, the three complementary techniques that were used indicate that the specific sequence of labeling intensity that we observed at the immunogold level reflects a *bona fide* developmental profile. Thus, immunofluorescence, immunoblots, and qRT-PCR concurred in showing early expression of laminin and agrin. The data also pointed to an abatement of laminin and agrin expression after the first postnatal week. This expression profile of agrin and laminin is consistent with these proteins having an instructive role in establishing the EBJC.

In the case of laminin and agrin, we did not perform a quantitative analysis of the immunogold labelling. For these proteins, the quantitative data obtained with Western blots and qRT-PCR complement the qualitative data obtained with the immunogold approach.

As the expression level of several of the proteins normally present at the perivascular astrocyte endfeet is very low at P0, establishment of astrocyte polarization must depend on two factors: (1) increased postnatal synthesis of perivascular endfoot proteins, and (2) postnatal insertion and stabilization of the proteins in perivascular endfoot membranes.

In vitro evidence suggests that agrin plays a role in the establishment of astrocyte polarization. A recent study has shown that exposure to agrin isoforms leads to increased AQP4 expression, membrane localization and formation of orthogobnal arrays of particles (OAP) in wild type and agrin-null mouse astrocytes (Fallier-Becker et al. 2011). The same effect was achieved by replacing exogenous agrin with ECM purified from Engelbreth-Holm-Swarm (EHS) mouse sarcoma, where laminin, collagen IV, heparan sulfate proteoglycan (a class of molecules that includes agrin), and nidogen/entactin are the major components. Our study shows that expression of agrin is at its highest level in the first postnatal week, prior to the peak of AQP4 expression, supporting the idea that agrin stimulates expression of AQP4. We did not distinguish between the neuronal and endothelial isoforms of agrin and cannot say whether these isoforms play distinct roles. Of note, agrin-null astrocytes did express AQP4 and OAPs, though at lower levels than did wild type controls, indicating that AQP4 expression and OAP formation do not depend solely on the presence of agrin. This conclusion finds support in a recent analysis of Rauch et al. (2011). These authors demonstrated a residual pool of AQP4 following targeted deletion of endothelial agrin. This residual pool might be kept in place by laminin, as discussed below.

Laminin has been suggested to play an important role in membrane localization of AQP4 in astrocyte endfeet. In vitro studies have shown that laminin clusters AQP4 and Kir4.1 in astrocyte membranes, and stabilizes these proteins at the basolateral domain of epithelial Madine-Darby canine kidney (MDCK) cells (Guadagno and Moukhles 2004; Tham and Moukhles 2011). The laminin α chain comes in two major isoforms in brain: α-1 and α-2. The antibodies used in this study are raised against full-length laminin and hence cannot distinguish between the different isoforms. However, our qRT-PCR data indicate that the expression of α-1 leads the expression of α-2. α-1 is the laminin isoform that predominates at the pial surface while α-2 predominates perivascularly. This developmental profile is entirely consistent with an instructive role of laminin, as our data suggest that AQP4 appears at an earlier stage subpially than perivascularly.

The developmental profile of α-syntrophin closely mimics that of AQP4. This finding bolsters the increasing body of evidence pointing to α-syntrophin as the immediate anchor of the perivascular AQP4 pool (Amiry-Moghaddam and Ottersen 2003; Nagelhus and Ottersen 2013). Dystrophin and dystroglycan display yet another developmental profile. These proteins can be detected early in the postnatal phase, but are then rather diffusely distributed. Only at day 7 do they begin to show a distinct perivascular accumulation. This coincides with the initial expression of AQP4.

The most parsimonious explanation of our findings is that laminin and agrin cause AQP4 and α-syntrophin to aggregate at endfoot membranes, through recruiting dystrophin and β-dystroglycan to this membrane domain. Previous studies have demonstrated that both dystrophin and β-dystroglycan are required for perivascular expression of AQP4 (Frigeri et al. 2001; Noell et al. 2011).

We also included an analysis of the inwardly rectifying K^+^ channel Kir4.1. At the protein level, Kir4.1 showed a developmental profile similar to that of AQP4. Previously it has been shown that these two proteins are strictly colocalized in macroglial membranes (Nagelhus et al. 1999). However, Kir4.1 mRNA differs from AQP4 mRNA in showing a sharp increase with age. Pending data on Kir4.1 regulation and turnover we cannot explain this intriguing difference between AQP4 and Kir4.1.

To what extent are the present findings relevant for the human brain? A study by El-Khoury et al. (2006) shows that AQP4 positive perivascular endfeet appear between gestational weeks 19–22 in humans (El-Khoury et al. 2006). Given that term in mice corresponds to gestational day ~120 in humans with respect to brain development, the finding of El-Khoury et al. (2006) is in good agreement with the situation in mice in which AQP4 positive endfeet emerge during the first postnatal days. Further studies are needed to establish whether the junctional complex in humans mimics that in mice in regard to the order of appearance of its molecular constituents.

There is now a growing awareness that loss of astroglial polarization occurs in a number of pathophysiological conditions, including temporal lobe epilepsy, ischemia and Alzheimer’s disease (Eid et al. 2005; Frydenlund et al. 2006; Yang et al. 2011; Heuser et al. 2012; Alvestad et al. 2013). What these diverse conditions have in common is a deficiency in homeostatic mechanisms that can easily be attributed to defunct handling of K^+^ and/or water. Notably, each of these conditions is characterized by neuronal hyperexcitability and increased proneness to seizures. Given the results obtained in the present study, there is a need to consider the possibility that the extracellular matrix (ECM) is more directly involved in cerebral pathophysiology than previously believed. Specifically, a loss of the instructive role of laminin or agrin could figure prominently in neurological disease. Proteolytic activities in the extracellular space, such as metalloproteinase activation (Michaluk et al. 2007), could be an upstream factor. These issues merit further investigation.

## Conclusion

Taken together with previous in vitro data, the present in vivo analysis suggests that the molecular complex of the gliovascular interface is assembled through a specific sequence of events. The basal lamina proteins laminin and agrin appear first, and stabilize β-dystroglycan and dystrophin at endfoot membranes. AQP4 and Kir4.1 are then recruited to the dystrophin complex in amounts that continue to increase towards adulthood. The developmental profile of α-syntrophin mimics that of AQP4, consistent with the idea that α-syntrophin serves as the immediate anchor of this water channel. We conclude that the ECM is likely to play a pivotal role in the development of the molecular assembly at the gliovascular interface. The pathophysiological roles of ECM warrant closer examination.

## Electronic supplementary material

Below is the link to the electronic supplementary material.

Supplementary material 1 (TIFF 11169 kb)

Supplementary material 2 (DOCX 13 kb)
