# Supplementary figures and images for: Postnatal development of the molecular complex underlying astrocyte polarization
Source: Brain Struct Funct. 2014 Apr 29;220(4):2087–101. doi: 10.1007/s00429-014-0775-z (PMC4481305; doi:10.1007/s00429-014-0775-z)

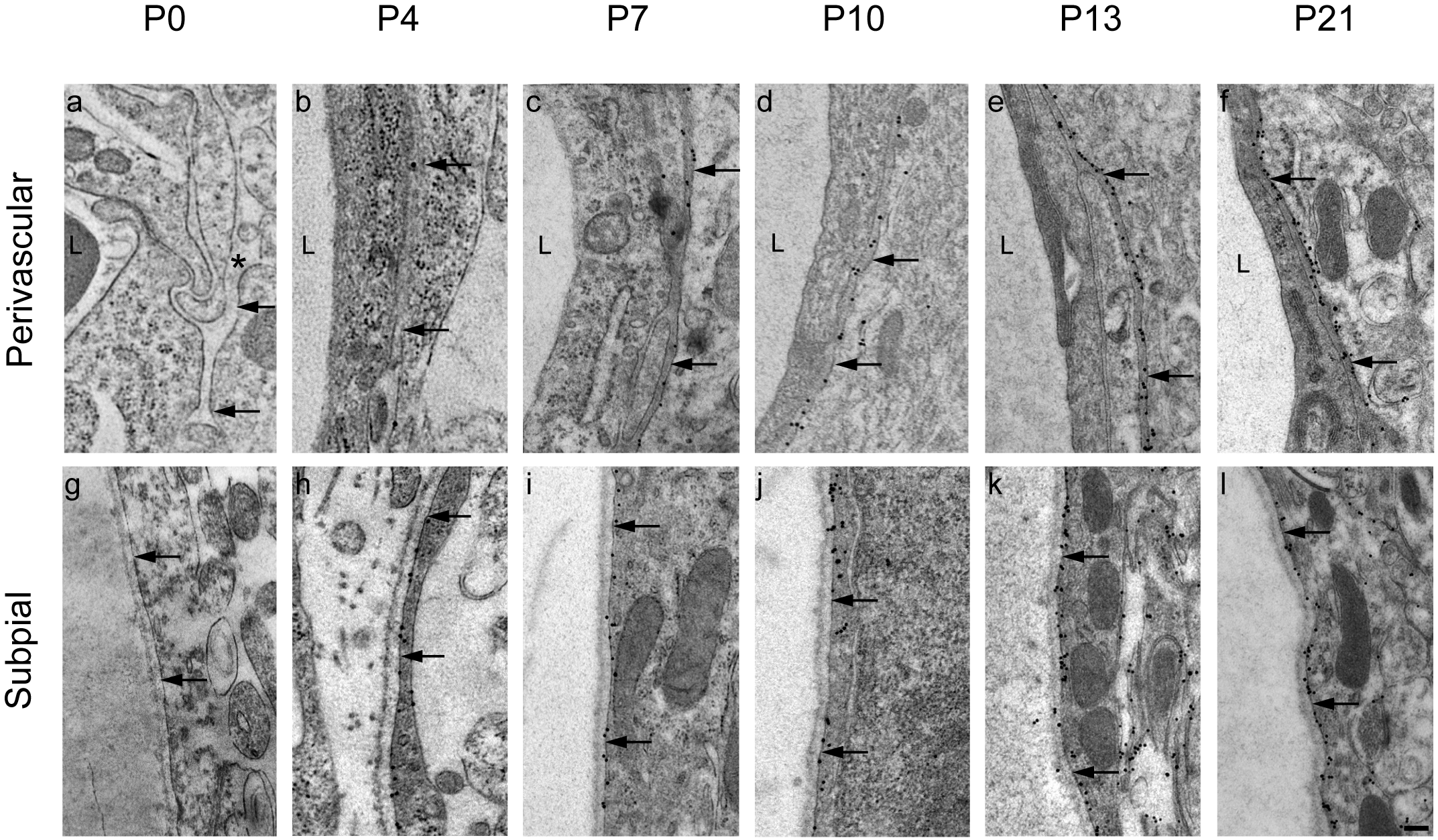

Supplement: Supplementary file 1 — Supplementary material 1 (TIFF 11169 kb) [file 429_2014_775_MOESM1_ESM.tif]
