# Supplementary material for: Postnatal development of the molecular complex underlying astrocyte polarization
Source: Brain Struct Funct. 2014 Apr 29;220(4):2087–101. doi: 10.1007/s00429-014-0775-z (PMC4481305; doi:10.1007/s00429-014-0775-z)
Supplement: Supplementary file 2 — Supplementary material 2 (DOCX 13 kb) [file 429_2014_775_MOESM2_ESM.docx]

**Supplementary figure S1:**

**Immunogold analysis shows that subpial endfeet are the first to accumulate AQP4**
a-l) Postnatal immunogold labeling of AQP4 at the perivascular (a-f) and subpial (g-l) astrocyte membranes in mouse neocortex. Perivascular and subpial membrane domains are indicated by arrows. a) At P0, the pericapillary basal lamina (asterisk) is immature, and there is no perivascular AQP4 immunogold labeling. b) At P4 the basal lamina is distinct but there is very weak AQP4 immunogold labeling (unlike the subpial membrane shown in h). c) At P7, the basal lamina is distinct, and AQP4 appears in the perivascular membranes. d-f) The AQP4 immunogold density increases further at P10-P21. g-l) The subpial basal lamina is well developed alreay at P0, and is associated with distinct AQP4 immunogold labeling in subpial astrocyte membranes. At P13 and P21, the AQP4 labeling extends into the glial lamellae beneath the pial surface. E, endothelial cells; L, vessel lumen; A, astrocyte (Scale bar: 200 nm).
